# Supplementary material for: Exploring socio-demographic determinants of breast self-examination practices among Jordanian women: insights from the 2023 population-based survey
Source: PLoS One. 2026 May 6;21(5):e0347331. doi: 10.1371/journal.pone.0347331 (PMC13148678; doi:10.1371/journal.pone.0347331)
Supplement: Attachment — Submitted filename: BSE (REVIEWED MANUSCRIPT).docx [file pone.0347331.s001.docx]

**Exploring Socio-Demographic Determinants of Breast Self-Examination Practices Among**

**Jordanian Women: Insights from the 2023 Population-Based Survey**

**Strengths of the Manuscript**

**Strengths of the Manuscript**

1. **Timely and Relevant Topic**
   - **Breast cancer is a significant public health concern, and the study provides valuable information regarding determinants of BSE practice in Jordan.**
   - **The focus on socio-demographic determinants is relevant to informing intervention design and increasing screening behavior.
     Large Sample Size**
   - **The study has the strength of having a nationally representative sample (12,304 married women) that ensures generalizability of findings**
2. **Use of JDHS Data**
   - **The study is founded on a well-established and trustworthy dataset, contributing to the validity of its findings.**
3. **Multivariate Analysis**
   - **The use of logistic regression is appropriate for the examination of different factors influencing BSE practices.**

**Areas for Improvement**

**1. Methodological Concerns**

- **Cross-Sectional Design Limitation**
  - **The study relies on a cross-sectional dataset, and causal inferences are partial. This must be articulated more explicitly under the limitations section.**
- **Potential Biases in Self-Reported Data**
  - **Since BSE practices were self-reported by the participants, recall bias and social desirability bias may affect response validity. Discussion of validation measures or consideration of this limitation more explicitly would strengthen the study**
- **Confounding Variables**
  - **While adjustments were made for several socio-demographic and behavioral variables, other possible confounders such as family history of breast cancer, access to health care, and cultural beliefs were not considered. Adjustment for these variables may strengthen the findings.**

**2. Clarity in Data Presentation**

- **Table Organization and Interpretation**
  - **The paper presents a great deal of statistical data, yet some tables and results must be more clearly organized for readability.**
  - **Consider bolding significant findings or summarizing the most critical points in a discussion section**
- **Multicollinearity Considerations**
  - **Some of the independent variables, such as education level and wealth index, are very likely to be highly correlated. It would strengthen the analysis to indicate whether multicollinearity diagnostics were conducted.**

**3. Discussion and Interpretation of Findings**

- **Comparisons with Previous Research**
  - **While the paper refers to earlier studies, there is room to compare more intently with similar research done elsewhere beyond Jordan to place findings**
- **Cultural and Behavioral Explanations**
  - **The paper mentions cultural barriers to BSE uptake but does not discuss at length specific cultural beliefs that may discourage self-examination. Further explanation on this issue would provide more insight**
- **Implications for Public Health Interventions**
  - **Although recommendations are given, real-world applications to healthcare providers, policymakers, and NGOs in Jordan must be more clearly outlined**

**4. Ethical Considerations**

- **Ethical Approval and Consent**
  - **The study used secondary JDHS data that is in the public domain. However, a more explicit explanation of ethical clearance procedures followed by the JDHS would facilitate transparency**

**Recommendations for Improvement**

1. **Strengthen the discussion on limitations, particularly for cross-sectional design and self-reporting data.**
2. **Describe methodological details, e.g., how multicollinearity was accounted for in regression models.**
3. **Discuss regional and cultural determinants of BSE practices to provide more meaningful contextual information.**
4. **Provide easier-to-read tables and figures to make data interpretation easier.**
5. **Make recommendations stronger by providing concrete actionable steps for healthcare providers and policymakers.**

**Conclusion**

**The manuscript makes an important contribution to the understanding of BSE practice among Jordanian women. Nevertheless, the resolution of methodological concerns, clarification of data presentation, and enhancement of cultural and policy-related discussions will substantially strengthen the manuscript's clarity and contribution.**

**Final Recommendation: *Major revisions required before acceptance for publication in PLOS ONE.***
