# Supplementary material for: Exploring socio-demographic determinants of breast self-examination practices among Jordanian women: insights from the 2023 population-based survey
Source: PLoS One. 2026 May 6;21(5):e0347331. doi: 10.1371/journal.pone.0347331 (PMC13148678; doi:10.1371/journal.pone.0347331)
Supplement: Attachment — Submitted filename: Response to Reviewers (2).docx [file pone.0347331.s002.docx]

Dear Editor/s

We sincerely thank the editor(s) and reviewers for their valuable and thoughtful insights regarding our research. We believe that the revision process helped us to enhance the clarity of our message and strengthened the overall quality of the manuscript. All comments have been carefully addressed in the following point-by-point responses.

**Academic Editor comments**

**Comment:** Please ensure that your manuscript meets PLOS ONE's style requirements, including those for file naming.

**Reply:** We understand the importance of adhering to the journal’s style requirements. Accordingly, we carefully revised our manuscript in line with PLOS ONE’s guidelines and made the necessary adjustments to ensure full compliance. We also reviewed all file names to confirm that they follow the required formatting

**Comment:** Please amend your list of authors on the manuscript to ensure that each author is linked to an affiliation. Authors’ affiliations should reflect the institution where the work was done (if authors moved subsequently, you can also list the new affiliation stating “current affiliation:….” as necessary)

**Reply:** We thank the editor for the clarification. We have carefully amended the authors list to ensure that it follows the mentioned criteria. All authors are affiliated with the institutions where the work was done, and no recent changes in their affiliations have occurred.

**Comment:** We note that Figure 1 in your submission contain [map/satellite] images which may be copyrighted.

**Reply:** We acknowledge the editor’s concerns regarding copyright issues for Figure 1. OpenStreetMap permits the public to copy, distribute, transmit, and adapt its data, provided that credit is given to OpenStreetMap and its contributors. In addition to including the required credit on the figure, we have revised the methods section and figure legend to explicitly reference OpenStreetMap as the source.

**Reviewer 1**

**Comment**: Cross-Sectional Design Limitation: The study relies on a cross-sectional dataset, and causal inferences are partial. This must be articulated more explicitly under the limitations section.

**Reply:** We truly appreciate the provided assessment and we would like to thank the reviewer for the effort and time spent to provide such extensive assessment. We acknowledge the reviewer comment and we understand that the current design does not provide causal relationships which is explicitly described in the limitations section:

“Being a cross-sectional study, it can only establish associations rather than causal links.”

**Comment:** Potential Biases in Self-Reported Data: Since BSE practices were self-reported by the participants, recall bias and social desirability bias may affect response validity. Discussion of validation measures or consideration of this limitation more explicitly would strengthen the study.

**Reply:** We thank the reviewer for this comment and we agree that the dataset was subjected to recall bias since BSE practices were self-reported. Therefore, recall bias is explicitly mentioned in the limitations section:

“Additionally, self-reported data on BSE practices may be subject to recall and social desirability biases. “

**Comment:** Confounding Variables: While adjustments were made for several socio-demographic and behavioral variables, other possible confounders such as family history of breast cancer, access to health care, and cultural beliefs were not considered. Adjustment for these variables may strengthen the findings

**Reply:** We appreciate the reviewer comment regrading the need to consider more confounding variables. However, since the available data was limited, we revised and edited the limitations section to discuss potential factors that could be considered confounders.

“The study also did not account for individual-level factors, such as personal access to healthcare services and screening facilities, the quality of health education campaigns and previous contribution to health education campaigns. which could provide deeper insights into the regional disparities in BSE practices. Other factors that were not considered in the current study include family history of breast cancer, awareness of breast cancer warning signs and cultural beliefs.”

**Comment:** The paper presents a great deal of statistical data, yet some tables and results must be more clearly organized for readability.

**Reply:** We thank the reviewer for pointing out the need to reorganize some results and tables considering better readability. We revised and edited our results section to offer a more logical flow. The descriptive findings were revised to start with age and progresses through marital status, parity, and pregnancy status, which are demographic and reproductive characteristics. Education and employment were then summarized followed by wealth to emphasize socioeconomic status. Regional and urban/rural variables were finally described to offer the geographic context. Tables 1 and 2 were also reorganized as, individual-level variables followed reproductive characteristics. Next, educational level, employment and wealth statuses. Then, media exposure took place and finally, regional variables were employed.

**Comment:** Consider bolding significant findings or summarizing the most critical points in a discussion section.

**Reply:** We thank the reviewer for this comment, as we believe it is important for scientific research reports to be clear and easy to read. The significant p-values are already highlighted in bold in Table 2. In addition, the discussion section has been revised to ensure that the most important findings are presented at the beginning, followed by a clearer explanation of what this manuscript adds to the existing literature:

“This study represents the first effort to examine the factors influencing BSE performance among adult women in Jordan using nationally representative data of 2023. Jordan is a country where breast cancer constitutes the most common cancer among females and the leading cause of cancer-related mortality in females [5]. Our analysis demonstrated a strong association between age and BSE performance, with older women significantly more likely to perform BSE. Women aged 45–49 were found to be three times more likely to practice BSE compared to those aged 20–24. These findings are consistent with studies by Al-Rifai et al. in Jordan 2015[18], Okyere et al. in Namibia 2023[20], and Wassie et al. in Kenya 2024[26]. This trend could be attributed to older women being more prone to health issues and more frequent interactions with healthcare providers[27].

The current analysis included data retrieved from the most updated JPFHS which allowed to investigate the long-term effect of the JBCP on the practice of BSE among Jordanian women [9]. Among the studied independent variables, media-related variables were considered, including internet and television, which provides novel insights on the effect of digital and televised campaigns which was previously underexplored.”

**Comment:** Multicollinearity Considerations: Some of the independent variables, such as education level and wealth index, are very likely to be highly correlated. It would strengthen the analysis to indicate whether multicollinearity diagnostics were conducted.

**Reply:** We thank the reviewer for highlighting this important point. We did not perform formal multicollinearity diagnostics in this analysis. We acknowledge that some variables, such as education level and wealth index, may be correlated, which could influence regression estimates. This has been added to the Discussion as a limitation.

**Comment:** While the paper refers to earlier studies, there is room to compare more intently with similar research done elsewhere beyond Jordan to place findings

**Reply:** We thank the reviewer for this comment and we acknowledge the importance of comparing results from Jordan to findings beyond Jordan, especially other countries with low-to-middle income. In fact, one advantage of DHS-based studies is that it follows standard methods that would allow for caparisons with previous or future research. We indeed compared the current results from Jordan to previous findings from Jordan, Egypt and Kenya and Namibia which provides the discussion with the needed context and depth as recommended by the reviewer

**Comment:** Cultural and Behavioral Explanations: The paper mentions cultural barriers to BSE uptake but does not discuss at length specific cultural beliefs that may discourage self-examination. Further explanation on this issue would provide more insight.

**Reply:** We thank the reviewer for this comment regarding cultural norms as barriers to BSE practice. We agree with the reviewer that these barriers may constitute a major concern toward, not only BSE practice, but also toward research in this field. We reorganized the cultural barriers previously stated in the introduction section to follow a more thematic structure as following:

“Barriers to BSE in Jordan include cultural, structural, and informational challenges [17]. Cultural norms, such as embarrassment, patriarchal family dynamics, and the need for male approval, often restrict women’s autonomy in health-related decisions. Additionally, religious beliefs, stigma, fatalistic attitudes, and reliance on traditional medicine diminish the perceived importance of screening.”

Although we did not restate the list of cultural barriers within the discussion section to avoid redundancy within the manuscript which may negatively impact readability, we revised and edited study limitations to offer a more extensive discussion of how these cultural norms and family dynamics can potentially lead to response bias and self-selection bias:

“Moreover, cultural barriers and reluctance to discuss sensitive health issues, including breast-related issues, may have led to response bias, potentially affecting the accuracy of the results. These cultural barriers can vary between urban and rural areas, where social norms and family dynamics are often more established in the rural areas. In addition, women who get exposed to health education campaigns may be more willing to respond to such survey which indicates the possibility of self-selection bias.”

**Comment:** Implications for Public Health Interventions: Although recommendations are given, real-world applications to healthcare providers, policymakers, and NGOs in Jordan must be more clearly outlined

**Reply:** We appreciate the reviewer comment and we agree that it is necessary to outline real-world applications within the recommendations section. Therefore, we added a call for healthcare providers to educate their patients and guide them regarding BSE practices:

“We encourage healthcare providers to educate their patients on BSE practice and provide guidance for them during their interactions with the healthcare system, particularly routine check-ups and family planning visits.”

We also added a call for governmental, non-governmental and community-based organization to participate actively in tailoring targeted initiatives to serve populations that show lower BSE rates:

“Lastly, tailored interventions in regions like Irbid and Mafraq, where BSE rates are lower, could address cultural and regional differences through localized awareness campaigns. The JBCP initiatives can play a major role in tailoring such interventions; however, community-based organizations and non-governmental organizations (NGOs) can also have significant contributions to this field. Primary healthcare centers in these areas should be advised to strengthen breast cancer health education initiatives to increase awareness about screening, including BSE.”

**Comment:** Ethical Approval and Consent: The study used secondary JDHS data that is in the public domain. However, a more explicit explanation of ethical clearance procedures followed by the JDHS would facilitate transparency

**Reply:** We appreciate the reviewer recommendation to elaborate on ethical considerations, including ethical approval and consent. We revised and edited the methods section to add a new subsection concerned with the ethical considerations of the current research work, including that’s of the JPFHS report and DHS program:

“The DHS program provided ethical approval for the JPFHS before the Jordanian Department of Statistics started data collection. Informed consent was a prerequisite for an individual to participate in the survey. The current study constituted a secondary analysis of de-identified data of JPFHS 2023. The DHS program provided the data and approved the research proposal of the present work. Given the anonymized nature of data, no additional ethical approval was required to analyze it.”

**Comment:** Strengthen the discussion on limitations, particularly for cross-sectional design and self-reporting data.

**Reply:** We appreciate the reviewer comment and we understand the importance of stating limitation of the research work to avoid overstating conclusions. In addition to limitations of the cross-sectional design and self-reported data, we also added the limited data on many other variables that may be considered as confounders. Also, discussion of response bias, self-selection bias and cultural barriers was added to the limitations section:

“However, there are several limitations to consider. Being a cross-sectional study, it can only establish associations rather than causal links. Additionally, self-reported data on BSE practices may be subject to recall and social desirability biases. The study also did not account for individual-level factors, such as personal access to healthcare services and screening facilities, the quality of health education campaigns and previous contribution to health education campaigns. which could provide deeper insights into the regional disparities in BSE practices. Other factors that were not considered in the current study include family history of breast cancer, awareness of breast cancer warning signs and cultural beliefs. Moreover, cultural barriers and reluctance to discuss sensitive health issues, including breast-related issues, may have led to response bias, potentially affecting the accuracy of the results. These cultural barriers can vary between urban and rural areas, where social norms and family dynamics are often more established in the rural areas. In addition, women who get exposed to health education campaigns may be more willing to respond to such survey which indicates the possibility of self-selection bias. If women who were more familiar with the concept of BSE were the primary respondents with underrepresentation of those who show limited awareness, this may contribute to the observed non-significant association between the type of residence and BSE practice.”

**Comment**: Describe methodological details, e.g., how multicollinearity was accounted for in regression models.

**Reply:** We thank the reviewer for this comment. Multivariable logistic regression was performed using SPSS as described. Multicollinearity was not formally assessed in this analysis. This has been noted as a limitation in the Discussion section.

**Comment**: Discuss regional and cultural determinants of BSE practices to provide more meaningful contextual information.

**Reply:** We thank the reviewer for this comment and we agree that it is important to discuss regional and cultural determinants of BSE. We elaborated more on regional disparities and variations in social norms and family dynamic between rural and urban areas in the discussion section. Also, we revised and edited our limitations section to add more details regarding how these disparities may lead to potential response and self-selection biases in the studied dataset:

“Moreover, cultural barriers and reluctance to discuss sensitive health issues, including breast-related issues, may have led to response bias, potentially affecting the accuracy of the results. These cultural barriers can vary between urban and rural areas, where social norms and family dynamics are often more established in the rural areas. In addition, women who get exposed to health education campaigns may be more willing to respond to such survey which indicates the possibility of self-selection bias. If women who were more familiar with the concept of BSE were the primary respondents with underrepresentation of those who show limited awareness, this may contribute to the observed non-significant association between the type of residence and BSE practice.”

**Comment**: Provide easier-to-read tables and figures to make data interpretation easier.

**Reply:** We thank the reviewer for this comment and we agree that the tables need reorganization. Therefore, we revised and rearranged the tables to follow a logical order as following: individual-level variables followed reproductive characteristics. Next, educational level, employment and wealth statuses. Then, media exposure took place and finally, regional variables were employed.

**Reviewer 2:**

**Comment:** Dear authors, I congratulate you on your work. Although it is written in a clear and understandable language, the subject discussed in the article is not a current issue. There are many studies in the literature on the subject. It would be more appropriate to publish it in a local journal.

**Reply:** We sincerely appreciate the reviewer’s positive feedback regarding the clarity and readability of our manuscript. We acknowledge the reviewer comment regarding the novelty of the current research work. We would like to respectfully clarify that the current study uses using the most recent 2023 Jordan Demographic and Health Survey (JDHS) dataset, which has not been explored yet in terms of BSE. We believe that it is important to reinvestigate such topic in the light of the public health programs employed by the Jordanian Breast Cancer Program (JBCP). Additionally, despite controversy regarding the value of practicing BSE, the national Jordanian breast cancer screening and diagnosis guidelines encouraged women to practice BSE which we outlined in the revised introduction section:

“While the national Jordanian breast cancer screening and diagnosis guidelines acknowledges the controversy regarding BSE, it encouraged women to practice BSE between routine screening to facilitate early detection of breast changes [12].”

Also, we believe that the current manuscript provides valuable insights to the international scientific community. Since the dataset was originally collected across Jordanian governates, which is a low-to-middle income country. The Jordanian efforts in breast cancer screening and early detection is an experience that worth being delivered to other low-to-middle income countries which rely heavily on low-cost awareness strategies, including BSE. Moreover, being derived from a representative DHS dataset, the current study offers standardized methods that allow for more future cross-country comparisons.

**Reviewer 3:**

**Comment**: The study benefits from a large sample size, appropriate statistical methods, and a clear presentation of findings. The manuscript is generally well organized, with adequate linkage to existing literature and a focus on practical recommendations. However, certain sections would benefit from improved clarity, more detailed interpretation of unexpected or non-significant findings, and slight enhancements in language and structure. Additionally, expanding the discussion around the implications of the results—particularly in relation to healthcare access, media outreach, and regional disparities—would strengthen the overall impact of the paper.

**Reply:** We thank the reviewer for the detailed and comprehensive assessment of our manuscript. We believe that such revisions improve scientific research quality and impactfully help researchers to deliver their findings to the scientific community. We carefully addressed the detailed assessment and responded to comments in a point-by-point frame as described below.

**Comment (Abstract):** The abstract effectively presents the study’s background, methods, key results, and conclusions. It communicates the public health relevance of BSE practices and offers clear insights into socio-demographic disparities. To further enhance its impact, consider including one or two statistical values to support your findings, revising minor grammatical issues, and clarifying the specific statistical method used.

**Reply:** We thank the reviewer for the detailed assessment of the abstract. We believe that this assessment helped us to better organize the abstract and enhance the presentation of our work. We carefully addressed all comments on the abstract and responded to them in a point-by-point frame as described below.

**Comment (Abstract):** While the methods section of the abstract provides sufficient information about the dataset and sample characteristics, please consider briefly mentioning the specific statistical method used (e.g., logistic regression), as this strengthens methodological transparency.

**Reply:** We thank the reviewer for the comment and we acknowledge the importance of specifying the statistical methodology within the abstract. We revised and edited the methods section within the abstract to add a sentence that explicitly states the statistical method:

“Associations between these variables and BSE were assessed using multivariable logistic regression.**”**

**Comment (Abstract):** The results are clearly presented and aligned with the study’s objectives. Nevertheless, including at least one adjusted odds ratio (AOR) and p-value in the abstract would enhance the precision and credibility of the reported findings.

**Reply:** We appreciate the reviewer comment and we agree that mention AORs and p-values enhance the precision and credibility of the abstract. We revised and edited the abstract to describe examples from the significant categories of age, education and wealth as following:

“Multivariate analysis revealed that signifcacnlty better BSE practice was observed among older women (e.g. age 45–49 vs 20–24: AOR 3.08, p < 0.001), those with higher education levels (e.g. secondary vs no education: AOR 2.41, p = 0.027), and wealthier women (e.g. richest vs poorest: AOR 1.54, p = 0.023).”

**Comment (Abstract):** The conclusion effectively highlights the importance of targeted interventions. You may consider briefly indicating potential implications for policy or future research directions in a concise sentence.

**Reply:** We truly appreciate this comment and we agree that it is helpful to direct future researchers based on the current findings. We previously recommended targeted health campaigns and culturally sensitive education. In addition, since we believe that cultural barriers are possible limitation to the current study, potentially leading to response bias and self-selection bias in the current sample, we finalized the conclusion with for future researchers to investigate such barriers as recommended by the reviewer:

“Future researchers are encouraged to further investigate cultural barriers toward BSE.”

**Comment (Abstract)**: The language is generally clear and concise. However, a few phrases could be revised for improved grammar and readability (e.g., ‘frequent reading newspaper of magazines’ → ‘frequent reading of newspapers or magazines’). A light copy-editing pass is recommended.

**Reply:** We thank the reviewer for notifying the issue in the phrase “frequent reading newspaper of magazines”. We edited the phrase to “frequent reading of newspapers or magazines” and subsequently screened the abstract for any language mistake and edited any captured mistake.

**Comment (Introduction):** The introduction provides a solid overview of the global and national burden of breast cancer and outlines the importance of BSE among Jordanian women. To further strengthen this section, I recommend clarifying the rationale for focusing on BSE despite evolving international recommendations, elaborating on how the present study differs from previous DHS-based analyses, and thematically organizing the discussion of barriers. These changes would improve the logical flow and enhance the justification for the study’s aims.

**Reply:** We sincerely appreciate the reviewer’s detailed assessment of the introduction section. We sincerely thank the reviewer for the time and effort spent to provide such comprehensive assessment. We provide a detailed, point-by-point response to the reviewer’s comments below.

**Comment (Introduction):** The manuscript opens with strong global breast cancer statistics, which provide important context. However, I recommend transitioning more swiftly from the global burden to the national (Jordanian) context to better anchor the reader in the geographic scope of the study.

**Reply:** We understand the reviewer’s concern regarding the potential redundancy in the reported global breast cancer statistics. Therefore, we revised this section and removed some of the global burden statistics to provide a more swifty transitioning to the national Jordanian statistics starting from the third sentence.

**Comment (Introduction):** The section effectively presents the prevalence and mortality rates of breast cancer in Jordan. To enhance its impact, consider briefly indicating whether national screening efforts (e.g., JBCP) have shown any progress in early diagnosis or survival rates since their implementation.

**Reply:** We thank the reviewer for highlighting the need to provide information about early diagnosis initiatives implemented by the Jordan Breast Cancer Program (JBCP). We believe that including details about the JBCP enriches the introduction section by providing better context on the status of breast cancer in the country. There has been a substantial increase in the reported incidence of breast cancer in Jordan. This increase likely reflects previously underdiagnosed cases rather than an actual increase in the incidence. We have incorporated this information into the introduction section as suggested by the reviewer. Additionally, we provided information regarding improvement in the overall 5-year survival among patients who were diagnosed after the establishment of JBCP.

“The incidence of breast cancer among Jordanian women has increased by 69% in 2015 compared to 2005. This increase is likely related to the screening and early diagnosis initiatives implemented by the Jordan Breast Cancer Program (JBCP) since its establishment in 2006 [6]. The overall 5-year survival rate was estimated at 64% among Jordanian women diagnosed with breast cancer between 1997 and 2002 [7]. In contrast, those diagnosed between 2011 and 2014, following the establishment of the JBCP, showed an improved 5-year overall survival rate of 84% [8].”

In the next paragraph, we further highlighted the proportion of patients diagnosed at an advanced stage of the disease before the establishment of the JBCP to complement the previously described more recent data:

“Before the establishment of the JBCP, more than 70% of patients were diagnosed at an advanced stage of the disease [9]. Unfortunately, a significant proportion of breast cancer cases in Jordan are still diagnosed at advanced stages. Recent data reveals that 36% of patients are diagnosed at stage III, and 14.1% at stage IV, meaning over half of cases are detected at late stages”

**Comment (Introduction):** While it is valid to highlight the affordability and accessibility of BSE, it should be noted that BSE is not strongly recommended in recent international screening guidelines. The authors are encouraged to justify their focus on BSE either by referencing Jordan-specific guidelines or explaining population-level barriers that make BSE particularly relevant in this setting.

**Reply:** We understand the reviewer comment regarding the international controversy regarding BSE. In addition to affordability and accessibility, we added the recommendations of the JBCP regrading BSE which encouraged women practice BSE to gain familiarity with their breasts and further can report any changes to a healthcare provider.

**“**While the national Jordanian breast cancer screening and diagnosis guidelines acknowledges the controversy regarding BSE, it encouraged women to practice BSE between routine screening to facilitate early detection of breast changes [12].”

**Comment (Introduction):** The manuscript refers to Al-Rifai et al. (2012) to establish a gap in the literature. While this is appreciated, it would strengthen the argument to more clearly articulate what this study offers that previous work has not—such as the inclusion of behavioral or media consumption variables, or an emphasis on regional disparities.

**Reply:** We truly thank the reviewer for this valuable suggestion. We have strengthened the argument of the research gap by emphasizing the growing use of digital and televised methods in awareness campaigns as understudied area.

“Also, the growing implementation of digital and televised awareness warrants further investigation.”

Further comparison between our research work and the previously published studies is provided in the discussion section to avoid redundancy in the current manuscript.

**Comment** **(Introduction):** The discussion of barriers—including cultural, religious, and economic factors—is comprehensive. To improve readability, consider organizing these barriers thematically (e.g., structural, informational, cultural) rather than listing them consecutively.

**Reply:** We true value the reviewer’s suggested thematic classification for BSE barriers. We revised and edited the classification to be more readable as suggested by the reviewer:

“Barriers to BSE in Jordan include cultural, structural, and informational challenges. Cultural norms, such as embarrassment, patriarchal family dynamics, and the need for male approval, often restrict women’s autonomy in health-related decisions. Additionally, religious beliefs, stigma, fatalistic attitudes, and reliance on traditional medicine diminish the perceived importance of screening. Structural barriers include financial constraints related to the absence of health insurance and high screening costs as well as accessibility challenges, such as the distance to healthcare facilities. Informational barriers include limited education, fear of diagnosis, lack of motivation, and misconceptions about cancer further discourage BSE [17].”

**Comment (Methods):** Add details on the sampling design and timeframe of data collection.

**Reply:** We understand the importance of mentioning sampling design and frame. We revised and edited the methods section to add a new subsection summarizing the sampling methodology of the Jordan Population and Family Health Survey (JPFHS) 2023:

“Representativeness of the Jordanian populations was a major consideration for the JPFHS; therefore, they adopted a two-stage stratified cluster sampling approach. The first stage constituted of the selection of primary sampling units (PSUs) based on population size and place of residence (urban or rural). In the second stage, households within each PSU were randomly selected. The Jordanian Department of Statistics implemented the survey between January and June 2023. The detailed sampling methodology is described in original JPFHS 2023 report [19].”

**Comment (Methods):** Improve justification for variable categorization.

**Reply:** We thank the reviewer for the comment and agree about the importance of explaining the rationale behind variable categorization. We aimed to follow the guidelines of DHS in order to keep consistency with earlier published DHS-based studies. We revised and edited the methods section to explain our rationale, provided with the corresponding citation:

We thank the reviewer for this comment. The categorization of independent variables in our study followed the DHS coding schemes and was provided as such in the dataset. Using these pre-defined categories ensures consistency with DHS guidelines and comparability with other DHS-based studies. We have clarified this point in the Methods section.

“All these categorizations were pre-defined by the DHS and provided as such in the dataset, ensuring consistency with DHS coding schemes and comparability with other DHS-based studies.”

**Comment (Methods)**: Elaborate on weighting and model diagnostics.

**Reply:** We thank the reviewer for this comment. Data were analyzed using SPSS version 24 with weighted counts applied according to DHS guidelines, using the sample weight provided in the dataset and adjusted by dividing by 1,000,000. Descriptive statistics summarized the study population, and multivariable logistic regression was conducted to identify factors associated with BSE practices, with results reported as adjusted odds ratios and 95% confidence intervals.

**Comment (Methods):** Include a brief ethical statement confirming secondary use of Demographic and Health Surveys (DHS) data.

**Reply:** We agree with the reviewer about the necessity to describe the ethical statement. We edited the methods section as recommended to add details about the approval from DHS program:

“A formal research proposal was submitted to the Demographic and Health Surveys (DHS) program website in order to access the dataset and gain approval to use the publicly available, de-identified, dataset for research purposes.”

Additionally, we added a new subsection within the methods section that is concerned with ethical considerations:

“The DHS program provided ethical approval for the JPFHS before the Jordanian Department of Statistics started data collection. Informed consent was a prerequisite for an individual to participate in the survey. The current study constituted a secondary analysis of de-identified data of JPFHS 2023. The DHS program provided the data and approved the research proposal of the present work. Given the anonymized nature of data, no additional ethical approval was required to analyze it.”

**Comment (Methods):** The authors state that the study utilized data from the 2023 Jordan Population and Family Health Survey (JPFHS). While this is appropriate, I recommend including the date of data collection (e.g., months/season) and a brief description of the sampling design (e.g., stratified, two-stage cluster sampling) for transparency and replicability.

**Reply:** We agree with the reviewer that discussion of sampling methodology would improve transparency and replicability of the current research work. Hence, we revised and edited the methods section to add a new subsection summarizing the sampling methodology of the JPFHS 2023 report.

“Representativeness of the Jordanian populations was a major consideration for the JPFHS; therefore, they adopted a two-stage stratified cluster sampling approach. The first stage constituted of the selection of primary sampling units (PSUs) based on population size and place of residence (urban or rural). In the second stage, households within each PSU were randomly selected. The Jordanian Department of Statistics implemented the survey between January and June 2023. The detailed sampling methodology is described in original JPFHS 2023 report [19].”

**Comment (Methods):** The definition of the dependent variable is clear; however, the phrase “breast cancer screening” may be misleading since the outcome is specifically related to Breast Self-Examination (BSE), not clinical or imaging-based screening. Please revise this terminology for precision.

**Reply:** We truly appreciate the reviewer comment about the term “breast cancer screening”. We agree with the reviewer about the need for a more precise term that describes the dependent variable; therefore, the term was revised to “breast self-examination practice”

**Comment (Methods):** The inclusion and exclusion criteria are appropriate. That said, the rationale for limiting the sample to ever-married women could be briefly explained. For example, were unmarried women excluded due to data availability, cultural reasons, or screening relevance? This clarification would help readers understand potential biases in sample selection.

**Reply:** We thank the reviewer for this comment. The sample was limited to ever-married women because the DHS dataset did not provide data for women who were never married, as shown in Table 1. Therefore, only ever-married women could be included in the analysis of breast self-examination practices. This rationale has been clarified in the Methods section.

**“**The analysis was limited to ever-married women because the DHS dataset did not include breast self-examination data for women who were never married, as shown in Table 1. Therefore, only ever-married women were included in the study.”

**Comment (Methods):** The range and categorization of independent variables are comprehensive and justified. However, some categorization decisions (e.g., for internet use and BMI) could benefit from referencing previous studies or DHS methodology to support the choices made.

**Reply:** We thank the reviewer for this valuable comment. We have now clarified in the Methods section that the categorization of variables such as internet use and media exposure followed the standard classifications provided by the DHS dataset. Additionally, we have updated the manuscript to explicitly state that BMI categorization was based on CDC criteria, which was not previously specified. This clarification has now been incorporated.

“Body mass index (BMI) was categorized as underweight, normal weight, overweight, and obese according to CDC criteria [25]”

**Comment (Methods):** The use of weighted logistic regression is appropriate for DHS data. The description of weighting is technically accurate but could be made clearer for broader audiences. I suggest briefly explaining why weighting is necessary in DHS analysis and specifying which weight variable was used.

**Reply:** We thank the reviewer for this helpful suggestion. In response, we have clarified the rationale for applying weighting in DHS analysis and have specified the weight variable used. A brief explanation was added to enhance clarity for readers. Weighted analyses were conducted using the women’s individual sample weight (v005), which accounts for survey design, sampling probabilities, and non-response to ensure nationally representative estimates. This information has now been incorporated into the Methods section.
“Weighted analysis was applied in accordance with DHS guidelines to ensure national representativeness, accounting for the complex sampling design, sampling probabilities, and non-response. The women's individual sample weight (v005), provided as an eight-digit variable with six implied decimal places, was divided by 1,000,000 for application in the analysis.”

**Comment (Methods):** It would be helpful to mention whether any multicollinearity diagnostics were performed before multivariable modelling, and whether model fit or goodness-of-fit statistics were assessed.

**Reply:** We appreciate the reviewer’s observation. Multivariable logistic regression was conducted as described; however, formal multicollinearity diagnostics and model fit/goodness-of-fit statistics were not performed in this analysis. This has now been acknowledged as a limitation.

**Comment (Methods):** Ethical Considerations: Although the authors mention that the data is publicly available, it would be beneficial to explicitly state that ethical approval and informed consent were handled by the original DHS program, and that no additional ethical clearance was required for secondary analysis.

**Reply:** We truly appreciate the reviewer suggestion to discuss the ethical consideration of the original DHS program. We revised our methods section to add a subsection for ethical considerations:

“The DHS program provided ethical approval for the JPFHS before the Jordanian Department of Statistics started data collection. Informed consent was a prerequisite for an individual to participate in the survey. The current study constituted a secondary analysis of de-identified data of JPFHS 2023. The DHS program provided the data and approved the research proposal of the present work. Given the anonymized nature of data, no additional ethical approval was required to analyze it.”

**Comment (Results):** The descriptive findings are informative; however, the presentation would benefit from a more structured flow. The transition between different variable groups (e.g., socio-demographic, behavioural, regional) appears abrupt. Grouping these variables more clearly in the narrative would enhance readability and comprehension for the reader.

**Reply:** We thank the reviewer for pointing out the need for better flow in presenting the descriptive findings. We revised and edited our results section to offer a more logical flow as recommended. While we did not follow the exact recommended grouping approach explicitly, the revised results section starts with age and progresses through marital status, parity, and pregnancy status, which are demographic and reproductive characteristics, Education and employment were then summarized followed by wealth to emphasize socioeconomic status. Regional and urban/rural variables were finally described to offer the geographic context.

**Comment (Results):** Table 1: While Table 1 provides a comprehensive overview of the sample characteristics, the accompanying text primarily reiterates values already shown in the table. The narrative should instead emphasize notable trends or meaningful contrasts that may not be immediately evident from the table alone. This would reduce redundancy and increase the analytical value of the results section.

**Reply:** We appreciate the reviewer comment about the repetitive statement of descriptive findings in Table 1 and the accompanying text. In addition to enhancing the flow of the accompanying text as recommended in a previous comment, we summarized and rephrased this section to highlight notable trends instead of just restating the values within the table. We believe that this comment indeed helped us to reduce redundancy and improve readability.

**Comment (Results):** Multivariate Analysis – Reporting of Significant and Non-significant Results: The logistic regression results are well reported, with appropriate use of adjusted odds ratios and confidence intervals. However, the text focuses predominantly on statistically significant findings, while non-significant associations are either overlooked or insufficiently addressed. Reporting both significant and non-significant outcomes strengthens transparency and helps to avoid potential bias in interpretation.

**Reply:** We thank the reviewer for this observation and we agree that non-significant associations are as important as significant ones. Indeed, the non-significant categories within categorized variables (e.g. age) were summarized while describing the variables. Also, a final statement of other non-significant variables is present at the end of results section:

“Our analysis revealed no significant associations between performing BSE and other factors, including current marital status, type of place of residence, BMI, current pregnancy status, employment status, frequency of watching television, or frequency of listening to the radio”

**Comment (Results):** Visual Data (Figure 1): The inclusion of a geographical distribution figure adds value to the manuscript. However, the figure is underutilized in the text. A more detailed interpretation of the observed regional differences and their potential public health implications would enhance the overall narrative and contextual relevance.

**Reply:** We thank the reviewer for this comment and we understand the importance of narratively describing regional differences. In the revised discussion section, regional differences are explicitly discussed in a detailed manner with provided implications and recommendations:

“Our analysis identified regional variations in BSE practices among women. Women residing in Balqa, Ajloun, and Aqaba demonstrated significantly higher BSE performance compared to those in Amman. Conversely, women from Irbid and Mafraq exhibited significantly lower BSE performance. No significant differences in BSE practices were observed among women in other governorates. These variations may be influenced by factors such as differences in health education outreach, cultural norms, socioeconomic status, and accessibility to healthcare services across regions. The nationwide efforts of the JBCP were intended to eliminate the geographical disparity, which has persisted since 2007[38]. Such disparities can manifest as lower healthcare infrastructure and fewer educational initiatives. Addressing these disparities requires tailored public health strategies that consider the unique characteristics and needs of each region to enhance BSE awareness and practice uniformly across the country. The efforts of the JBCP in eliminating healthcare-related disparities are manifested as a lack of significant associations between urban or rural residence and BSE practice. The findings of the current study can be considered as a basis to focus on governates that showed lower BSE practice. We also recommend conducting further research to gain a deeper understanding of the variations in BSE practice across different regions.”

**Comment (Discussion):** The authors present a good summary of the main results at the beginning of the discussion. However, it would be helpful to briefly restate why these findings are important for public health in Jordan.

**Reply:** We thank the reviewer for pointing out the importance of restating the context of the current research work in the beginning of the discussion. We added a sentence that describes the burden of breast cancer in Jordan within the first paragraph of the discussion section:

“Jordan is a country where breast cancer constitutes the most common cancer among females and the leading cause of cancer-related mortality in females”

**Comment (Discussion):** The manuscript compares its findings with previous studies from Jordan and other countries, which is appreciated. The discussion could be improved by explaining more clearly how this study adds new knowledge to the existing literature.

**Reply:** We thank the reviewer for this comment which helped us to highlight how the current study adds to the existing literature. In addition to the previously stated strengths in the “strengths and limitations” section, we added a paragraph after the opening paragraph that provides insights about “what is new” in the current research and what readers should expect next in the discussion section:

“The current analysis included data retrieved from the most updated JPFHS which allowed to investigate the long-term effect of the JBCP on the practice of BSE among Jordanian women [9]. Among the studied independent variables, media-related variables were considered, including internet and television, which provides novel insights on the effect of digital and televised campaigns which was previously underexplored.”

**Comment (Discussion):** Some results, such as the positive relationship between smoking and BSE, are surprising. The discussion mentions a possible reason, but it would be helpful to say that more research is needed to better understand this finding.

**Reply:** We thank the reviewer for this valuable comment and we agree that the positive relationship between smoking and BSE is indeed surprising. In addition to the comparison with previous findings and suggesting a possible reason, we added a recommendation for future research to further investigate this area:

“However, future researchers are encouraged to further research the effect of smoking status on the attitudes toward BSE to provide more robust evidence.”

**Comment (Discussion):** The authors found that BSE practices vary across regions in Jordan. This is important. However, the reasons for these differences are not fully explained. A short paragraph discussing possible causes (e.g., differences in health education or access to care) would be useful.

**Reply:** We appreciate the reviewer comment and agree that disparities between geographic regions are important to be discussed. We edited the previously stated discussion on health disparities in the manuscript to elaborate more on theses disparities, adding more depth to this area:

“Our analysis identified regional variations in BSE practices among women. Women residing in Balqa, Ajloun, and Aqaba demonstrated significantly higher BSE performance compared to those in Amman. Conversely, women from Irbid and Mafraq exhibited significantly lower BSE performance. No significant differences in BSE practices were observed among women in other governorates. These variations may be influenced by factors such as differences in health education outreach, cultural norms, socioeconomic status, and accessibility to healthcare services across regions. The nationwide efforts of the JBCP were intended to eliminate the geographical disparity, which has persisted since 2007[38]. Such disparities can manifest as lower healthcare infrastructure and fewer educational initiatives. Addressing these disparities requires tailored public health strategies that consider the unique characteristics and needs of each region to enhance BSE awareness and practice uniformly across the country. The efforts of the JBCP in eliminating healthcare-related disparities are manifested as a lack of significant associations between urban or rural residence and BSE practice. The findings of the current study can be considered as a basis to focus on governates that showed lower BSE practice. We also recommend conducting further research to gain a deeper understanding of the variations in BSE practice across different regions.”

**Comment (Discussion):** Some factors (e.g., BMI, place of residence, television watching) were not related to BSE, but the discussion does not mention them. Even if they are not significant, it is good to briefly comment on why this might be the case.

**Reply:** We appreciate the reviewer comment pointing out the need to discuss non-significant results to avoid any bias during results interpretation. We added a new paragraph to the discussion section that briefly discuss non-significant association as following:

“Several factors, including BMI, type of residence (urban vs. rural), marital status, current pregnancy status, employment status, and frequency of watching television or listening to the radio. BMI showed non-significant association with BSE practice which suggests that body weight does not influence women attitude toward BSE. The non-significant associations with marital status, pregnancy, and employment may indicate that BSE practice is driven by individual awareness and education rather than these variables. Moreover, the non-significant association between watching television or listening to the radio and performing BSE can be explained by the increasing reliance on digital media.”

A new sentence was added to the discussion of the geographic regions to address the non-significant association between the type of residence and BSE practice:

“The efforts of the JBCP in eliminating healthcare-related disparities are manifested as a lack of significant associations between urban or rural residence and BSE practice”

**Comment (Discussion):** The limitations section identifies relevant issues such as the cross-sectional design and self-reported nature of BSE data. However, it would be useful to expand on the limitation that the study did not include some potentially important individual-level variables, such as: family history of breast cancer, knowledge of breast cancer warning signs, access to screening facilities, prior participation in health education programs.

**Reply:** We thank the reviewer for pointing and we agree that it is necessary to describe them in the limitations section in addition to the previously stated factors. We revised and edited the limitations of the study to add these variables:

“The study also did not account for individual-level factors, such as personal access to healthcare services and screening facilities, the quality of health education campaigns and previous contribution to health education campaigns. which could provide deeper insights into the regional disparities in BSE practices. Other factors that were not considered in the current study include family history of breast cancer, awareness of breast cancer warning signs and cultural beliefs”

**Comment (Discussion):** The mention of cultural barriers is appropriate, but a clearer explanation of how such factors may vary by region (or urban/rural residence) would make this limitation more meaningful.

**Reply:** We appreciate the reviewer recommendation to add more explanations on how cultural barriers are considered a limitation of the current research work. We revised the limitations section to add details on how cultural barriers could influence the findings, taking the type of residence into consideration:

“Moreover, cultural barriers and reluctance to discuss sensitive health issues, including breast-related issues, may have led to response bias, potentially affecting the accuracy of the results. These cultural barriers can vary between urban and rural areas, where social norms and family dynamics are often more established in the rural areas. In addition, women who get exposed to health education campaigns may be more willing to respond to such survey which indicates the possibility of self-selection bias. If women who were more familiar with the concept of BSE were the primary respondents with underrepresentation of those who show limited awareness, this may contribute to the observed non-significant association between the type of residence and BSE practice.”

**Comment (Discussion):** The recommendation to use digital media and newspapers is logical; however, it might be useful to add a brief note on the accessibility of these media among older or rural women.

**Reply:** We thank the reviewer for pointing out the need to acknowledge the limited access to digital platforms among older women and those who reside rural areas. We revised and edited the limitations section to add a brief note on this point as recommended:

“Utilizing media, especially the internet and newspapers, can increase BSE awareness, particularly for digitally connected women. However, conventional community-based health education campaigns remain necessary to outreach older women and those who reside rural areas since these two populations may have limited access to digital platforms.”

**Comment (Discussion):** Outreach to nulliparous women is a valuable point, and it may be strengthened by briefly mentioning how health providers (e.g., during family planning or general check-ups) could play a role in this outreach.

**Reply:** We truly appreciate this comment which helped us to provide direct recommendations to the healthcare provides. We revised our recommendations section to add a call for healthcare professionals to educate and guide their patients in terms of BSE during patients’ interactions with the healthcare system:

“Outreach to nulliparous women could help bridge the gap in BSE performance, as multiparous women showed higher adoption rates. We encourage healthcare providers to educate their patients on BSE practice and provide guidance for them during their interactions with the healthcare system, particularly routine check-ups and family planning visits.”

**Comment (Discussion):** The call for region-specific awareness efforts is important. This could be improved by briefly suggesting which stakeholders (e.g., local health departments, NGOs) could help implement such programs.

**Reply:** We thank the reviewer for this comment and we agree that different governmental and non-governmental organizations should contribute significantly and impactfully to such tailored initiatives. We revised and edited the recommendations section to add a call for governmental, non-governmental and community-based organizations to contribute to these initiatives:

“Lastly, tailored interventions in regions like Irbid and Mafraq, where BSE rates are lower, could address cultural and regional differences through localized awareness campaigns. The JBCP initiatives can play a major role in tailoring such interventions; however, community-based organizations and non-governmental organizations (NGOs) can also have significant contributions to this field. Primary healthcare centers in these areas should be advised to strengthen breast cancer health education initiatives to increase awareness about screening, including BSE.”

**Comment (Conclusion):** The conclusion accurately reflects the key findings of the study and provides a concise summary. However, it would be helpful to rephrase the sentence: “frequent reading newspaper of magazines” to “frequent reading of newspapers or magazines” for clarity and correctness.

**Reply:** We appreciate the reviewer comment which helped to improve consistency and readability of the conclusion. We rephrased the sentence from “frequent reading newspaper of magazines” to “frequent reading of newspapers or magazines” as advised by the reviewer.

**Comment (Conclusion):** While the statistical findings are summarized, the conclusion may be slightly strengthened by including one sentence on the potential public health value of improving BSE practices (e.g., increased early detection, reduced late-stage diagnosis).

**Reply:** We thank the reviewer for this comment and we understand the importance of describing public health value. We previously mentioned in the conclusion that: “Targeted interventions focusing on younger women, those with lower education, and women in economically disadvantaged regions could help increase BSE adoption and improve early breast cancer detection.”. However, we believe that highlighting a potential increased early detection and reduced late-stage diagnosis as a direct consequence of BSE practice would be considered an overstatement of the findings since the current research did not analyze a dataset that allows for such conclusion.

**Comment (Conclusion):** Ending with a brief call for future research—such as follow-up studies, interventions, or qualitative exploration of cultural barriers—would add depth and signal future directions.

**Reply:** We truly appreciate this comment and we agree that it is helpful to direct future researchers based on the current findings. Since we believe that cultural barriers are possible limitation to the current study, potentially leading to response bias and self-selection bias in the current sample. We finalized the conclusion with for future researchers to investigate such barriers as recommended by the reviewer:

“Future researchers are encouraged to further investigate cultural barriers toward BSE.”
