# Supplementary material for: Exploring socio-demographic determinants of breast self-examination practices among Jordanian women: insights from the 2023 population-based survey
Source: PLoS One. 2026 May 6;21(5):e0347331. doi: 10.1371/journal.pone.0347331 (PMC13148678; doi:10.1371/journal.pone.0347331)
Supplement: Attachment — Submitted filename: Response to Reviewers.docx [file pone.0347331.s003.docx]

Dear Editor in Chief:

We truly appreciate the time and effort spent by you and the reviewers. We have carefully addressed the reviewers’ comments in a point-by-point format below. We hope that our revised manuscript will be satisfactory for publication.

Best wishes.

**Reviewer #1:**

**Comment:** Congratulations on addressing all the comments spelled out in the manuscript. You have done a great work, and I recommend that the manuscript should be accepted for publication.

**Reply:** We thank the reviewer for the positive feedback on the revised manuscript and appreciate the positive recommendation. We truly appreciate the time you spent to provide a comprehensive assessment to the manuscript during the peer review process.

**Reviewer #3:**

**Comment:** Thank you for your careful and thoughtful revisions. The revised manuscript is much improved in both clarity and coherence. Your incorporation of previous reviewer feedback is appreciated, and your study continues to offer valuable insights into predictors of Breast Self-Examination (BSE) behavior among Jordanian women.

**Reply:** We thank the reviewer for the kind words and the provided comprehensive assessment to the manuscript. We have carefully addressed the comments and the recommendations in a point-by-point format below.

**Comment (Terminology Consistency):** Please ensure consistent use of the term “Jordan Population and Family Health Survey (JPFHS)” throughout the text. The abbreviation “DHS” is used in some places—this should be harmonized to avoid confusion.

**Reply:** We thank the reviewer for this comment which helped us improve consistency and avoid confusing readers. We revised the manuscript and revised Jordan Demographic and Health Survey (DHS) into Jordan Population and Family Health Survey (JPFHS) wherever it was meant to refer to the Jordanian dataset. Particularly, lines: 39, 40, 111 and 179.

**Comment (Grammar):**

Original: “reading newspaper or magazines”

Suggested: “reading newspapers or magazines”

**Reply:** We appreciate the reviewer for this comment and agree that the phrase needed grammatical correction in some parts of the manuscript. We revised the phrase to “reading newspapers or magazines” as recommended in Table 2 and lines 58 and 474.

**Comment (Discussion Section – Clarification):**

Consider rephrasing the sentence summarizing non-significant findings to improve clarity:

“Several factors, such as BMI, residence type, marital status, and pregnancy history, were not significantly associated with BSE in the final regression model.”

**Reply:** We thank the reviewer for this comment since it helped us improve the discussion of the non-significant findings. We revised the sentence to: “Several factors, such as BMI, residence type, marital status, employment status and pregnancy history, were not significantly associated with BSE in the final regression model”.

**Comment (Table Titles):**

Table 1: You may consider refining the title to:

“Sociodemographic characteristics by BSE status”

Table 2:

“Multivariate logistic regression results for predictors of BSE”

**Reply:** We thank the reviewer for this recommendation. We revised the titles to “Sociodemographic characteristics by BSE status” and “Multivariate logistic regression results for predictors of BSE” for Tables 1 and 2, respectively, as suggested by the reviewer.

**Comment (Punctuation):**

Please ensure consistency in the use of commas in lists throughout the manuscript (e.g., “education level, internet use, and newspaper reading”).

**Reply:**

We thank the reviewer for this comment. lists were revised all over the manuscript to ensure consistency in the use of commas. It was refined particularly in lines 7, 46, 240, and 409.

**Comment:** Overall, the manuscript is strong and requires only these minor adjustments. No further revisions to content or analysis are needed.

**Reply:** We thank the reviewer for these comments which are necessary for improving the rigor and clarity of our manuscript. We believe that the revised version addressed these minor adjustment, offering a more clear and readable manuscript.
